# Homœopathy and Hydropathy Out-done by the Te-to-tum Doctor of Arkansas

**Published:** 1848-01

**Authors:** 


*Homœopathy and Hydropathy out-done by the Te-to-tum Doctor of Arkansas.*—An intelligent friend from the South informs us that there is in Arkansas a Doctor who practices medicine upon an entirely new system—called the *te-to-tum system.* He employs an instrument, with eight sides, similar to the toy used by children and known as the te-to-tum, only larger in size and differently lettered. On each side of the octagon is a letter of the alphabet, designed to represent a certain thing—as, for example, V. for vomit, G. for glister, P. for purge, C. for calomel, &c., &c. Whenever this worthy is called to see a patient, he takes with him this wonder-working instrument, and without examining either the pulse or the tongue, or asking any questions, proceeds to spin it, as one would a te-to-tum, and administers according to its revelations. Thus, if V. is turned up, he forthwith declares that an emetic is indispensable; if C. that calomel must be given, and so on.

The success which has attended this new plan of practice, we are told, is astonishing, and the popularity of its author, unbounded; so much so, as to cast homœopathy, hydropathy, and Eclectic-Thompsonianism, completely in the shade. This is something new and original in the way of quackery, and we see no reason why it should not have its day, as it is quite as philosophical as many other systems now in vogue. But if we might be permitted to make a suggestion to the lucky discoverer, whoever he may be, we would say that he might, perhaps, improve his plan by substituting some Egyptian, Chinese, or Arabian hieroglyphics for the plain English characters, which every one understands, as the "*enlightened public*" always yield their faith to a system in proportion as it is dark and mysterious, and to them incomprehensible.—*St. Louis Medical and Surgical Journal.*

